# Automated Assessment of Upper Extremity Movement Impairment due to Stroke

**DOI:** 10.1371/journal.pone.0104487

**Published:** 2014-08-06

**Authors:** Erienne V. Olesh, Sergiy Yakovenko, Valeriya Gritsenko

**Affiliations:** 1 Division of Physical Therapy, School of Medicine, West Virginia University, Morgantown, West Virginia, United States of America; 2 Division of Exercise Physiology, School of Medicine, West Virginia University, Morgantown, West Virginia, United States of America; University of Lethbridge, Canada

## Abstract

Current diagnosis and treatment of movement impairment post-stroke is based on the subjective assessment of select movements by a trained clinical specialist. However, modern low-cost motion capture technology allows for the development of automated quantitative assessment of motor impairment. Such outcome measures are crucial for advancing post-stroke treatment methods. We sought to develop an automated method of measuring the quality of movement in clinically-relevant terms from low-cost motion capture. Unconstrained movements of upper extremity were performed by people with chronic hemiparesis and recorded by standard and low-cost motion capture systems. Quantitative scores derived from motion capture were compared to qualitative clinical scores produced by trained human raters. A strong linear relationship was found between qualitative scores and quantitative scores derived from both standard and low-cost motion capture. Performance of the automated scoring algorithm was matched by averaged qualitative scores of three human raters. We conclude that low-cost motion capture combined with an automated scoring algorithm is a feasible method to assess objectively upper-arm impairment post stroke. The application of this technology may not only reduce the cost of assessment of post-stroke movement impairment, but also promote the acceptance of objective impairment measures into routine medical practice.

## Introduction

Fifty percent of stroke survivors suffer from an impairment of motor function that requires prolonged rehabilitation [1], [2]. Because the impairment of upper limb function is a predictor of long-term participation in activities of daily life [3] and quality of life post stroke [4], reduction of arm impairment is an important aspect of rehabilitation [5]–[7]. Rehabilitation programs for upper extremity are designed and delivered by physical or occupational therapists, based on their assessment of movement impairment. The success of this approach depends on the amount of experience and skillfulness of the therapist, and on the duration of treatment. However, there is no standard procedure for the assessment and treatment of the impairment in arm movement. This leads to the variability in the effectiveness of therapy and to the inability to compare interventions across practitioners and clinics. Furthermore, current consensus is that physical therapy continues to be effective months and years after a neurological damage, such as stroke [8]–[10]. However, with the current one-on-one hospital session approach, prolonged treatment is extremely expensive and usually does not last beyond the first month following a stroke. These limitations of current medical care create a strong motivation to deliver therapy at home [11]. Multiple home-based therapy systems are currently being developed world-wide [12]–[21].

To enable cross-evaluation of home-based treatments and help them move out of research realm into clinical practice, it is important to develop standard quantitative outcome measures that draw on the accumulated clinical experience of impairment assessment. The current state-of-the-art in clinical assessment of movement impairment is based on the subjective scoring of select movements by a trained clinical specialist. Several standard tests exist to assess the impairment of arm function, such as Fugl-Meyer Assessment (FMA) [22] and Action Research Arm Test (ARAT) [23] to name a few. These tests have established reliability, validity, and responsiveness values [24]–[29]. We propose to use validated clinical tests of movement impairment to develop an automated quantitative assessment of impairment. This will allow to not only standardize clinical impairment assessment, but also include it into home-based therapies and promote their cross-validation.

Recent technological improvements have resulted in low cost 3D motion capture systems such as Kinect Sensor (Microsoft). Such technology holds the potential of significantly advancing impairment assessment by providing objective kinematic data with which to guide the development of novel therapies (for review see [30]). Recent studies have shown that Kinect Sensor can be used to quantify clinically-relevant parameters of gait [31],[32] and posture [33], [34]. Kinect-based virtual stepping therapy has been shown to be effective for post-stroke rehabilitation of gait [35]. Several recent pilot studies have also demonstrated that Kinect-based motion capture helps motivate neurological patients to participate in physical therapy [36], and that such therapy is well received by both patients and therapists [37], [38]. However, quantitative assessment of arm impairment continues to be a challenge. To meet this challenge, we have developed the algorithm of automated clinical scoring for quantifying arm impairment. In this study we have tested this algorithm in its ability to quantify post-stroke upper extremity impairment from low-cost motion capture, and we compared its performance to that of trained human raters.

## Materials and Methods

West Virginia University Institutional Review Board approved the protocol entitled A New Quantitative Biomechanical Method for Motor Assessment of Disability number 1311129283. Prior to experiment, participants signed informed consent approved by the Institutional Review Board.

### Participants

Study participants were adults with chronic hemiparesis with the following characteristics: 4 female, 5 male, 58±21 years old, 5±6 years post-stroke (standard deviation, s.d., is stated after ± here and in the rest of the manuscript). They were medically stable and could comprehend simple instructions. Infarct locations were identified from MRI scans by the participant's care providers (Table 1). One subject was excluded from data analysis, because her self-report of stroke was not confirmed by her hospital chart.

**Table 1:** Summary of participant characteristics.

Participant	Age	Sex	Years post stroke	Dominant Hemisphere	Stroke Hemisphere	Stroke Location
**1**	50	Male	5	Right	Right	Caudal medulla
**2**	76	Male	2	Left	Right	Posterior globus pallidus and internal capsule
**3**	20	Female	20	Right	Right	Middle Cerebral Artery distribution involving portions of frontal and temporal lobes
**4**	80	Female	1	Right	Left	Posterior Limb of Internal Capsule
**5**	62	Male	2	Right	Right	Frontal intraparenchymal hemorrhage
**6**	39	Female	1	Right	Right	Middle Cerebral Artery distribution involving portions of frontal and parietal lobes, putamen, and globus pallidus
**7**	76	Male	4	Right	Left	Anterior temporal lobe and posterior left putamen
**8**	64	Male	4	Right	Left	Middle Cerebral Artery distribution involving portions of frontal lobe

### Procedures

**Figure 1:**
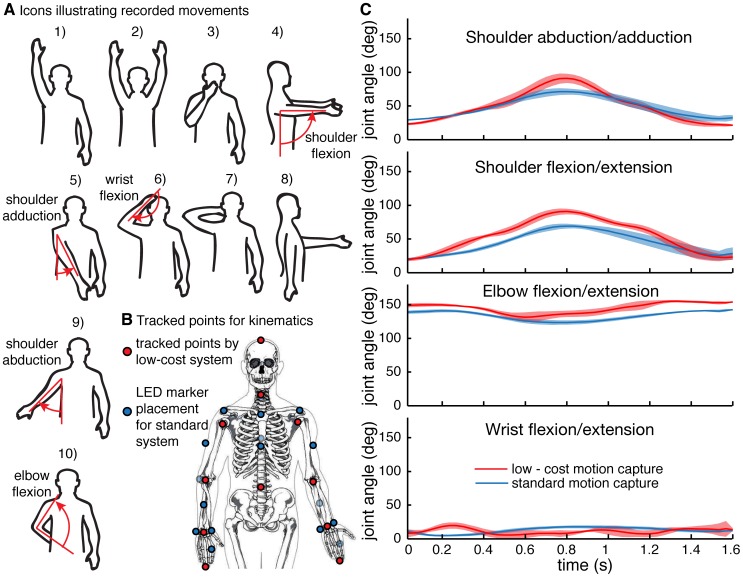
Study methodology. A, illustration of recorded movements. Red lines indicate the direction of motion for joint angles included in the kinematic analysis. B, tracked points used to calculate joint angles. C, Average joint angles of the left arm calculated from the data captured by the two motion capture systems during 10 repetitions of the movement 8) shown in A.

The participants performed 10 different arm movements (Fig. 1A) that are part of FMA [22] and ARAT [23]. The participants repeated each movement between 5 and 28 times after a demonstration by the experimenter. The movements were captured simultaneously by a standard motion capture system Impulse (Phase Space), the low-cost motion capture device Kinect Sensor (Microsoft), and recorded with a high-definition video camera (Samsung) for scoring by human raters. Movement selection was based on current capabilities of Kinect Sensor to track position of large arm segments, but not individual fingers.

Data were processed in Matlab (MathWorks, Inc.). The coordinates of multiple tracked skeletal landmarks were captured at 480 Hz by the standard system and at 30 Hz by the low-cost system (Fig. 1). These data were filtered using a second order Butterworth low-pass filter (cut-off at 6 Hz). Next, we calculated four joint angles (shoulder flexion/extension, shoulder abduction/adduction, elbow flexion/extension, and wrist flexion/extension; termed kinematics) from motion capture data recorded by both systems during a single repetition of each of the 10 movements performed by the non-paretic and paretic limbs. Joint angles reflect independent degrees of freedom of the arm and, thus, encompass complex information about movement limitations of people post-stroke.

The temporal alignment of the corresponding movements for paretic and non-paretic arms was accomplished in three steps. Firstly, movement start and end was manually identified in a subset of data. Secondly, kinematic data aligned on these onsets were averaged per joint angle to create a mean trace, termed wavelet, for each movement kind. Lastly, the multiple movements per trial were identified using peaks in the correlation coefficient profile for different delays between joint angles and the wavelet. The time of peaks were further used to align movement repetitions within and across trials. Manual creation of the wavelet can be omitted in a fully automated version of this analysis, if a single movement is recorded per trial.

### Estimating minimal number of movement repetitions for low-cost assessment

We have used kinematics recorded by both systems to estimate the minimal number of movement repetitions required for sufficiently precise motion capture with the low-cost system. To accomplish this estimation we bootstrapped the data in several steps to estimate errors of averaging one, two, three, etc repetitions of the same movement. The errors were absolute differences between the maximal amplitude of angular motion in a single trial and the maximal amplitude of average angular motion across all corresponding trails. The following steps were carried out to bootstrap these errors: 1) To estimated the error from **1** repetition of the same movement, single-trial errors were drawn repeatedly and randomly with replacement from the dataset for each movement type and each participant. The average squared differences between the mean error and each of the single-trial errors was the estimate of error of low-cost motion capture during a single movement. 2) To estimate the error from **2** repetitions of the same movement, two single-trial error values were drawn repeatedly and randomly with replacement from the dataset for each movement type and each participant. The average squared differences between the overall mean error and the mean of two single-trial errors was the estimate of error of low-cost motion capture after two repetitions of a movement. 3)–20) This bootstrapping was repeated with increasing number of trials (samples drawn from the population), until the maximal number of repetitions was reached for a particular movement and participant.

Lastly, we determined the first bootstrapped error value that fell below the 95% confidence interval of the mean error for each movement and participant. The corresponding number of trials used to calculate this value of error indicated the minimal number of repetitions of the same movement needed for accurate motion capture by the low-cost system.

### Principle Component Analysis (PCA) for automated scoring of impairment

**Figure 2:**
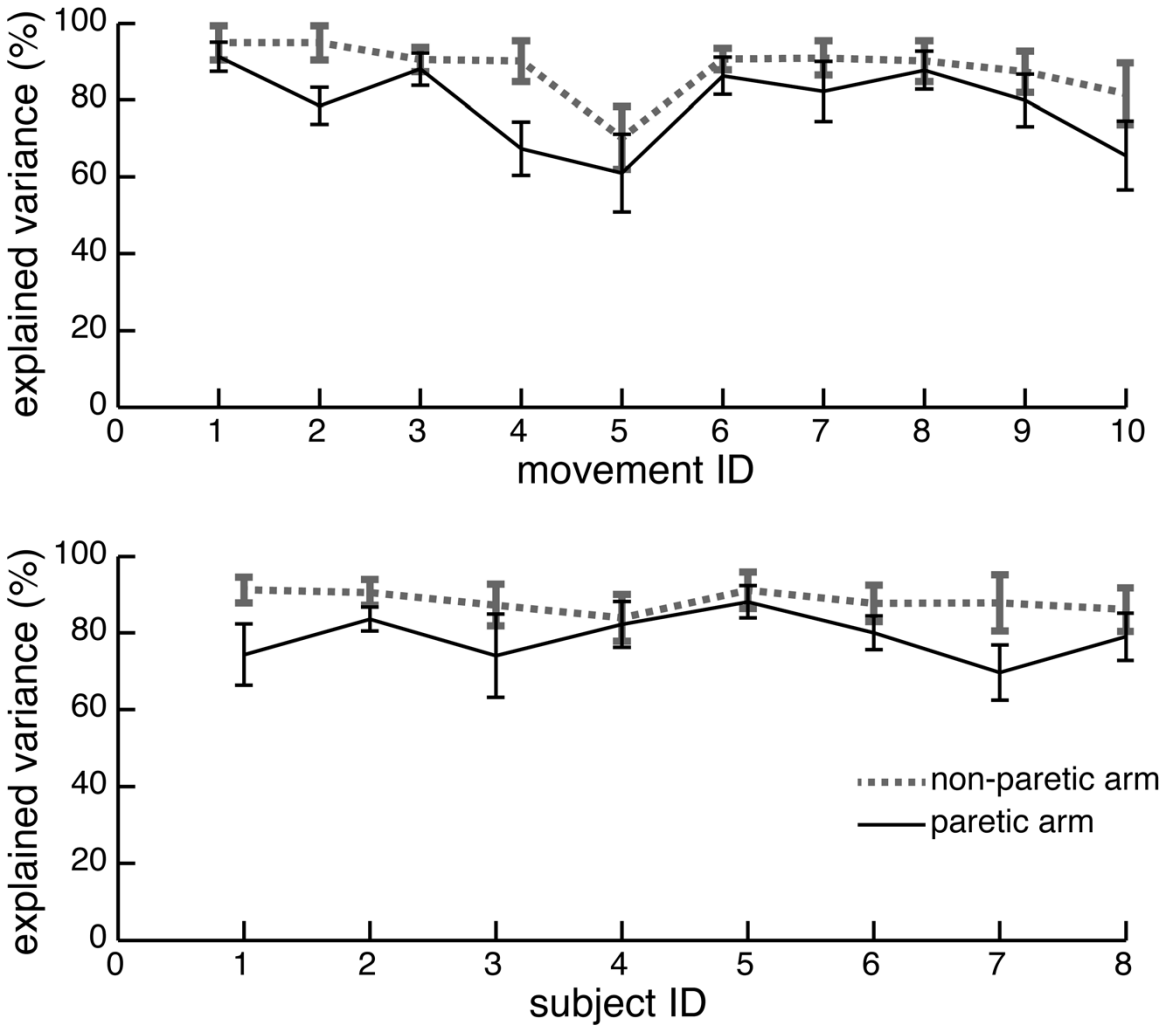
Results of principal component analysis. Cumulative explained variance and the number of principal components are shown for each movement type across participants (top plot) and for each participant across movement types (bottom plot). Grey dotted lines show results of decomposition of movement of the non-paretic arm, while black solid lines shows results of decomposition of movement of the paretic arm. The principal components were derived from mean data and used to reconstruct data from individual movements.

Joint angles of the non-paretic arm of each subject were averaged across repetitions of the same movement, and principal components were derived from the averaged temporal profiles across the four joint angles using eigenvalue decomposition of the covariance matrix. Then, individual temporal profiles of the joint angles of paretic arm recorded during each repetition of each movement were reconstructed with the basis of the principal components derived from the averaged profiles of non-paretic arm. The number of principal components chosen for the reconstruction were sufficient to explain ≧95% of variance in the kinematics. The reconstructed joint angle profiles were compared to the original paretic profiles using coefficient of determination (R^2^), which indicated how closely non-paretic principal components represent the movement of paretic arm. Thus, this measure constitutes ***a quantitative score of impairment*** (WVU ©2012). The same decomposition was done on non-paretic data from individual trials using the principal components derived from the averaged non-paretic data. This measure showed the inherent variability of scoring using this method. The resulting R^2^ values for both of these analyses are plotted in Figure 2.

The principal components were extracted from the demeaned joint angle profiles. The process of demeaning the data served to improve the quality of impairment assessment by removing inaccurate biases in the low-cost motion capture. Furthermore, the reduction of data dimensionality using principal component analysis also reduced the sensitivity of impairment assessment to noise in the low-cost motion capture data.

### Clinical scoring of impairment

Thirty graduate students in the last year of their Degree of Physical Therapy generated standard qualitative scores by rating 5 repetitions of each movement from video recordings of study participants. Movements were rated on the Fugl-Meyer scale, 0 indicating no movement at all, 1 indicating slow and/or abnormal movement, and 2 indicating normal movement [22]. Students were instructed to follow this scale to the best of their ability. Intraclass correlation coefficient for the relationship between the mean group scores and each rater's scores was used to establish inter-rater reliability [39].

The strength of the relationship between the quantitative scores derived from standard and low-cost motion capture and between the quantitative and qualitative scores was determined using linear regression. The power of the Pearson correlation coefficient (β) was determined from a statistical table [40]. Regression was also used to define the linear decoding model. The decoding performance of this linear model was evaluated by fitting regressions into data for all but one subject and then using this regression to predict the qualitative score of the subject that was left out. This was repeated for all 8 subjects.

### The number of raters that match performance of automated scoring

To estimate how many human rates it would take to match automated scoring performance, we bootstrapped the qualitative scores in several steps similarly to the procedure described above. The qualitative scores of 30 human raters and quantitative scores from low-cost motion capture were used for this analysis. The mean qualitative score averaged across all raters represents the most accurate clinical measure of a participant's impairment. The average squared differences between the mean qualitative scores and the qualitative scores of each rater was the estimate of error of individual human raters. The rest of the qualitative scores were bootstrapped using the following approach. To compare the error of 2 human raters to the automated performance, qualitative scores produced by 2 human raters were drawn repeatedly and randomly with replacement from the dataset of qualitative scores for each movement type and each participant. The drawn values were averaged, subtracted from the overall mean qualitative scores and squared. The resulting population of qualitative score errors represented the estimate of errors of 2 human raters. This bootstrapping was repeated with increasing number of raters (samples drawn from the population), until the maximal number of 30 raters was reached for a particular movement and participant.

Lastly, we determined the first bootstrapped qualitative score error value that fell below the model performance error for each movement and participant. The corresponding number of raters used to calculate the value of qualitative score error indicated the minimal number of human raters it would take to surpass performance of the automated scoring algorithm.

## Results

### Quality of movement assessment using low-cost system

**Figure 3:**
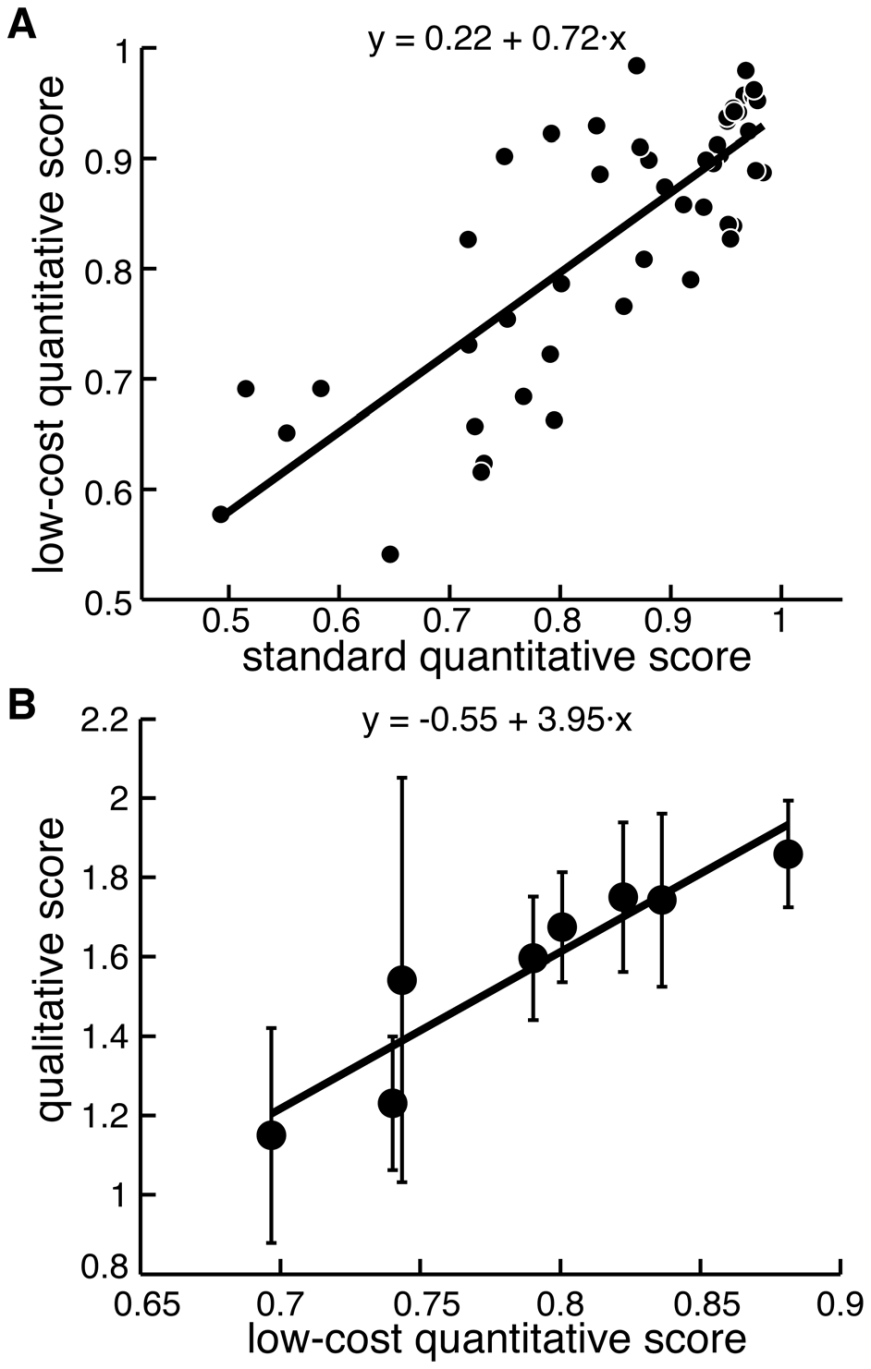
The comparison between quantitative scores from standard and low-cost motion capture and qualitative scores. A, Dots show mean scores for each movement and each subject; thick line shows a regression fit. B, Symbols show mean scores for each subject; error bars show s.d. across 10 movements; thick line shows a regression fit.

The quality of unconstrained 3D movements performed by each subject with paretic and non-paretic arm was automatically scored from kinematics. There was a strong linear relationship between the quantitative scores derived from both motion capture systems (p<0.001; R^2^ = 0.64; Fig. 3A), indicating that they are analogous.

The standard clinical tests usually evaluate performance of single repetitions of different movements. To test the feasibility of using low-cost motion capture for clinical testing, we have estimated how many repetitions of the same movements it would take to achieve accurate kinematic data from the low-cost system. The number of repetitions of the same movement needed to obtain a mean estimate that falls within the 95% confidence interval was 1.98±0.50 trials for shoulder abduction/adduction; 1.97±0.44 trials for shoulder flexion/extension; 1.88±0.34 trials for elbow flexion/extension; 1.85±0.48 trials for wrist flexion/extension. This makes it feasible to use low-cost motion capture for fast automated testing.

### Qualitative scores vs. quantitative scores

**Figure 4:**
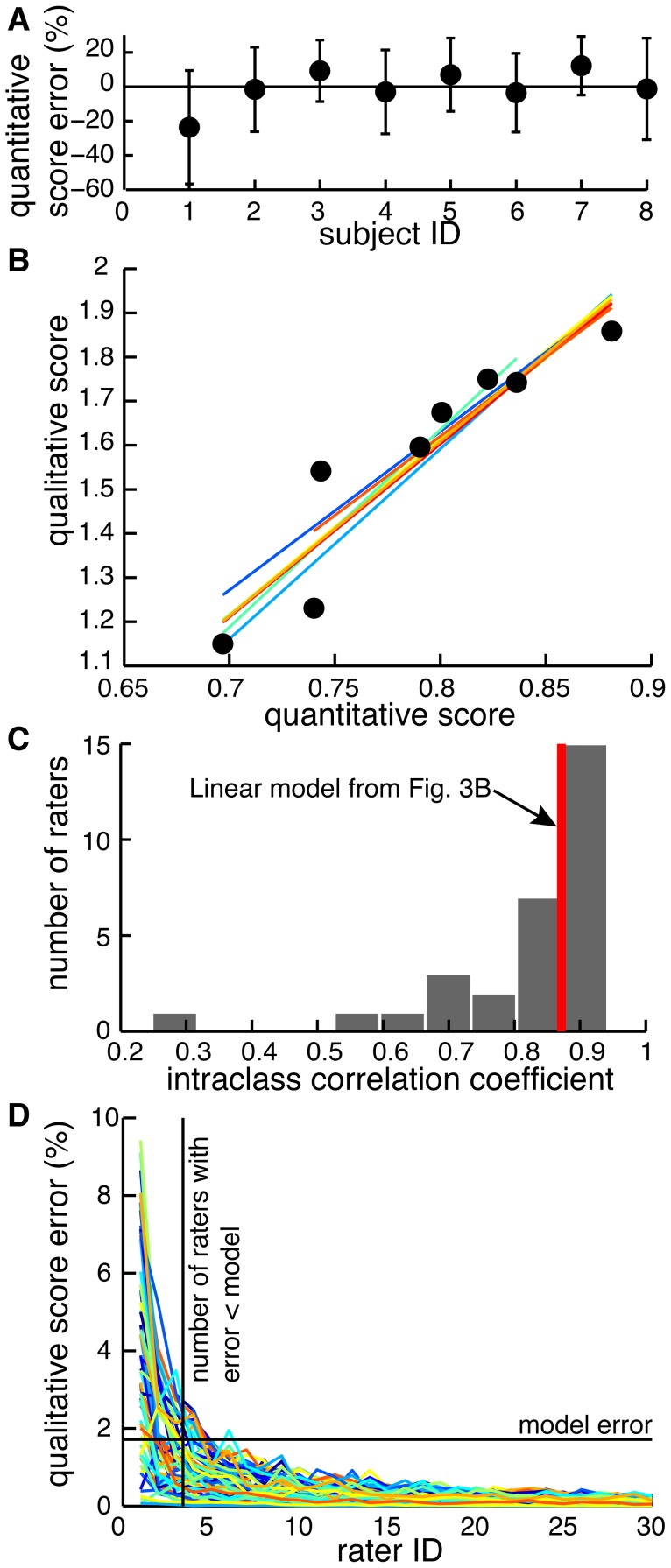
Decoding performance. A, Error in predicting each subject's qualitative score from regressions fitted to the rest of the participants. Mean errors are expressed as % of the correct score; error bars show s.d. across 10 movements. B, Symbols show the same data as in Fig. 3B; lines show regressions for datasets with one subject's data point removed. C, Histogram of intraclass correlation coefficients for relationships between individual human raters and the mean qualitative score. D, Colored lines show reducing errors as more raters score movements of the same participants per movement type, limb, and participant.

To score subject movements in clinically-relevant terms, we analyzed the motion capture data by converting it into physiological joint angles and applying PCA. More than 95% of variance across joint angles during the average movement of the non-paretic arm was represented by two principal components in all but one movement. These principal components could be used to reconstruct individual movements performed by both non-paretic and paretic arms with explained variances equal to 88.24±2.60% and 78.90±5.98% respectively. The quantitative scores based on the explained variances of paretic movements were linearly related to the qualitative scores (p = 0.001; β = 0.97) with R^2^ = 0.868 (Fig. 3B). The decoding performance of this linear model was characterized by the mean error of predicted scores being 7.68±7.52% of the maximal score (Fig. 4A). Regression offsets ranged from −1.94 to −1.24, slopes ranged from 3.58 to 4.46, and R^2^ ranged from 0.78 to 0.93 when individual participants were taken out of the dataset (Fig. 4B). This shows that it is feasible to automatically score movement impairment using low-cost motion capture.

### Consistency of human raters compared to quantitative scores

We have used the average scores of human raters as the gold standard against which to compare our automatic scoring algorithm. However, the accuracy of human raters varies due to the subjective nature of this approach. The proposed quantitative analysis offers an accurate and unsupervised alternative to the subjective and time-consuming measures. The tuned scoring model has a comparative reliability of combined scores from 30 human raters in our study (Fig. 4C). The algorithm used in this study performs as well as 3.42±1.78 human raters (s.d. is across movements; Fig. 4D). This further supports the feasibility of using motion capture for automated scoring of movement impairment.

### Variability of scoring across different test movements

**Figure 5:**
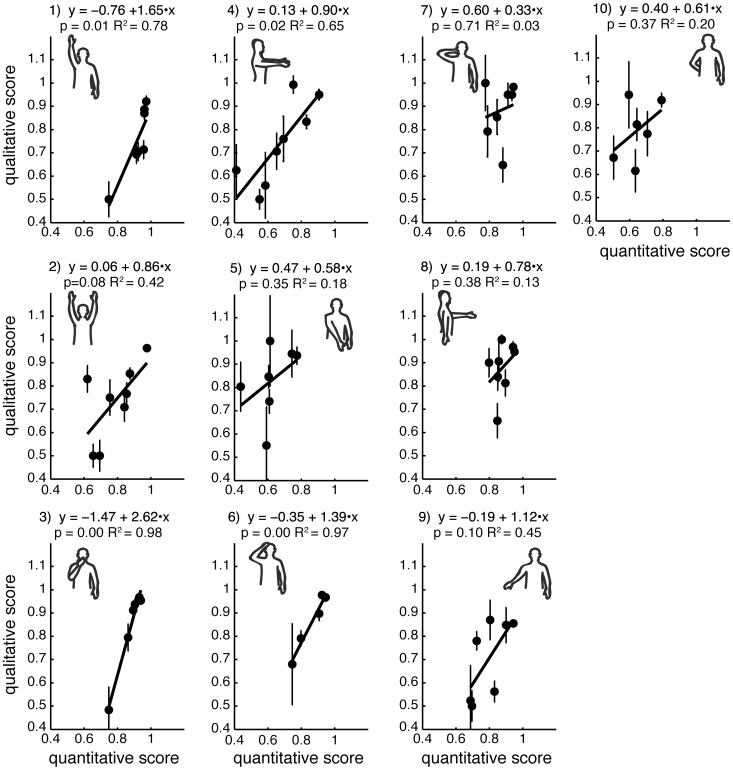
Relationships between qualitative and quantitative scores for each movement type. Dots show mean scores across participants, thick lines show linear regressions with their equations and fit statistics above each plot.

PCA has shown that different movements typically included in clinical tests have different inter-trial variability. This is illustrated by the changes in the explained variance of decomposition based on mean principal components between different recorded movements (Fig. 2, top plot). This variability translates into variability of the relationship between qualitative and quantitative scores for each movement (Fig. 5). This suggests that some of the movements included in clinical tests may provide less reliable information about movement impairment because of their high inter-trial variability. Nevertheless, all relationships between quantitative and qualitative scores had positive slopes. This further supports our conclusion that using low-cost motion capture for automated scoring of movement impairment is feasible.

### Accuracy of low-cost motion capture

The standard motion capture system was used as the gold standard to assess the kinematic accuracy of the low-end system. To compare the two systems we calculated the root mean squared (RMS) errors between them with single trials aligned on movement onset as described above. RMS errors were averaged across the duration of each movement and across the two limbs for each of the four physiological angles. In addition to the RMS errors, we have also calculated the absolute difference between maximal joint excursions for each movement captured by each of the systems. The mean errors of joint angles recorded by the low-cost motion capture system were considerable (Table 2). These errors are primarily due to biases, i.e. consistent over- or under-estimation of joint angles by the Kinect sensor due to inaccurate identification of tracked points on the body. Despite such large errors, quantitative assessment with PCA was successful in reproducing clinical assessment as shown above. This is because PCA is less sensitive to biases and noise in the motion capture data compared to RMS or movement excursion measures for reasons described above in the Methods section.

**Table 2:** Angular errors of low-cost motion capture relative to the standard system.

	shoulder abduction/adduction angle	shoulder flexion/extension angle	elbow flexion/extension angle	wrist flexion/extension angle
**Mean RMS errors, degrees**	22.03±9.55	25.81±10.57	22.88±8.15	15.99±7.41
**Mean RMS errors, % of max.**	12±5	14±6	14±5	18±8
**Mean maximal joint excursion error, degrees**	22.08±23.65	26.31±14.54	3.76±16.05	6.27±14.77
**Mean maximal joint excursion error, % of max.**	12±13	14±8	2±10	7±16

Table contains mean values ± standard deviations across participants and movements. Max. stands for maximal range of motion.

## Discussion

The study results have shown that using low-cost motion capture with an automated scoring algorithm is a feasible method to assess objectively upper-arm impairment post stroke. Several recent studies have demonstrated the usefulness of whole-body kinematics in the assessment of improvements in post-stroke locomotion [41], arm-trunk coordination [42], and reaching movements [43]. Furthermore, motion capture was used to assess upper extremity motor function after constraint-induced movement therapy and was reported to have higher inter-rater reliability than possible with traditional clinical measures [44], [45]. However, some major limitations of using motion capture for clinical needs is the cost, complexity, and lack of portability of traditional full body motion capture systems, which require several cameras and markers placed on subject's body. With the development of low-cost markerless 3D motion capture systems, such as the Kinect Sensor used in this study, out-of-the-lab movement kinematics with sufficient accuracy is now available for general use. The potential cost savings for clinics using the new low-cost motion capture technology are substantial, e.g. Kinect Sensor costs about $200, while lab-based motion capture systems cost tens of thousands of dollars. However, the complexity of kinematic data is still a barrier to the widespread acceptance of it in clinical practice. Results of the current study aim to overcome this barrier by demonstrating the effectiveness of an automated algorithm to clinically assess arm impairment from kinematics. This allows for the automation of impairment assessment, which enables the inclusion of quantitative outcome measures in routine medical practice. Clinical automated assessments are already a reality for quantitative measures of gait and balance impairment using GAITRite (CIR Systems Inc) and SMART Balance Master (NeuroCom) respectively. The current study is the first to show that clinical assessment of arm motor impairment can be automated. The application of this technology may not only reduce the cost of assessment of post-stroke movement impairment, but also promote the acceptance of objective impairment measures into routine medical practice.

Results of our study have shown that automated quantitative assessment of movement impairment was as reliable as clinical assessment by thirty senior DPT students. This is consistent with previous studies showing that using motion capture for clinical assessment results in increased inter-rater reliability [44], [45]. While inter-rater reliability between highly experienced therapist is likely to be higher, we believe that it is valid to compare automated performance against raters with variable levels of experience. This is because including raters with variable abilities is a more accurate representation of variance in skill in clinical practice. Overall, our results shows that automated scoring of motor impairment can increase the accuracy of clinical assessment. Furthermore, using a consistent algorithm for the analysis of kinematic data can help standardize outcome measures across medical specialists and across facilities.

Traditionally, clinical tests consist of different movements that are performed once by the patient. A single repetition of each movement is done to reduce the time it takes to perform the test, and thus reduce the time spent by a medical specialist on motor assessment. We have shown that to obtain reliable kinematics from Kinect Sensor, each movement has to be repeated three times. While this increases the time it takes for the patient to perform the test, averaging across repetitions of the same movements contributes to the increased reliability of motor assessment. Furthermore, the medical specialist will not need to be present during the test administration, thus his/her time spent on the assessment will be reduced. Therefore, we believe that it is feasible to implement the automated motor assessment in a clinical setting.

A limitation of the current study is that we employed a very coarse, although robust, 3-point clinical scale for the assessment of movement quality. Such scale has the resolution of 1/3 or 33% of maximal range of motion. Therefore our data show that while the low-cost motion capture system is less accurate than the laboratory standard, it is more accurate than the 3-point clinical scale (Table 2). Future studies are needed to test the effectiveness of the PCA-based quantitative assessment in presence of biases and noise in the low-cost motion capture for scales with higher resolution and for more complex movements involving the hand.

Assessment of motor impairment using the FMA is useful for understanding the limitations in motion of individual joints and basic synergy patterns. However, to evaluate the effectiveness of rehabilitation in enabling people to return to their normal lives different kinds of movements prove more useful. For example, clinical tests of functional abilities such as Wolf Motor Function Test [46], rely on movements that mimic goal-directed tasks of daily living, e.g. picking up or manipulating household objects. Therefore, the next logical step for the development of quantitative assessment based on low-cost motion capture is to evaluate its effectiveness to extract information about the individual's function from such goal-directed movements.
